# Initial validation of the Chinese version VIA Youth-96 and age-related changes in character strengths among adolescents

**DOI:** 10.3389/fpsyg.2022.906171

**Published:** 2022-10-11

**Authors:** Xiaotong Cheng, Shuang Xu, Yuyan Huang, Cheng Qin, Kezhi Liu, Mingyuan Tian, Xiaoyuan Liao, Xinyi Zhou, Bo Xiang, Jing Chen, Wei Lei

**Affiliations:** ^1^Department of Psychiatry, The Affiliated Hospital of Southwest Medical University, Luzhou, China; ^2^Laboratory of Neurological Diseases and Brain Function, The Affiliated Hospital of Southwest Medical University, Luzhou, China; ^3^Nuclear Medicine and Molecular Imaging Key Laboratory of Sichuan Province, Luzhou, China; ^4^The Second Veterans Hospital of Sichuan, Chengdu, China; ^5^School of Clinical Medicine, Southwest Medical University, Luzhou, China

**Keywords:** character strength, VIA Youth-96, age-related changes, adolescent, parental autonomy support, parental psychological control

## Abstract

This study aimed to preliminary examine the psychometric properties of the Chinese version 96-item VIA Inventory for Youth (VIA Youth-96) by analyzing the internal consistency, factorial validity, and criterion validity, and to examine the age-related changes in character strengths (CSs) among adolescents. The sample consisted of 959 adolescents aged 10–17 (49.5% boys). Participants completed the Chinese version VIA Youth-96, along with the Perceived Parental Autonomy Support Scale, and questionnaires assessing life satisfaction and self-efficacy online. The Chinese version VIA Youth-96 showed a good fit for the original four-factor structure, and CS scores were significantly correlated with life satisfaction and self-efficacy indicating a good criterion validity of the scale. The internal consistency was 0.54–0.86 for subscales. Moreover, this study revealed significant age-related changes in CSs among adolescents, eight CSs significantly linearly declined by age. These results suggested that the Chinese version VIA Youth-96 is a valid tool for assessing CSs in adolescents and that CSs are declined linearly by age during adolescence.

## Introduction

Identifying individual strengths of character and fostering them is crucial to positive youth development (Park, 2004). Character strengths (CSs) are a set of morally valued character traits that are critical to a good life (Peterson and Seligman, 2004) and mental health (Peterson and Seligman, 2004; McGrath, 2015). Consistent evidence showed that higher CSs are associated with a higher level of life satisfaction [a distinct construct representing a cognitive and global evaluation of the quality of one's life as a whole (Pavot and Diener, 2008)] (Park and Peterson, 2006; Ruch et al., 2014; Bruna et al., 2019; Martínez-Martí et al., 2020) as well as self-efficacy [an individual's belief in their capacity to execute behaviors necessary to produce specific performance attainments (Bandura, 1977)] (Ruch et al., 2014; Casali et al., 2021). Adolescents with a higher level of CSs are happier, do better at school, are more popular among peers, and have fewer psychological and behavioral problems (Park, 2009). For example, several studies showed that the love of learning and perseverance were particularly beneficial to a series of educational outcomes (Wagner and Ruch, 2015; Weber et al., 2016; Wagner et al., 2020). In addition, CSs were protective factors that allow for better resilience to stress and likely prevent depression (Padilla-Walker et al., 2020). Intervention programs that aimed at encouraging recognition and use of one's CSs increased life satisfaction, positive affect, classroom engagement, and even academic performance (Lavy, 2019).

The Values in Action (VIA) classification (Peterson and Seligman, 2004) defined 24 CSs, i.e., appreciation of beauty and excellence, bravery, creativity, curiosity, fairness, forgiveness, gratitude, honesty, hope, humility, humor, judgment, kindness, leadership, love, love of learning, perseverance, perspective, prudence, self-regulation, social intelligence, spirituality, teamwork, and zest. These CSs were organized into six core virtues, namely wisdom and knowledge, courage, humanity, justice, temperance, and transcendence. Based on the VIA classification, the VIA Inventory for Youth (VIA-Youth) has been developed to measure individual CSs for adolescents (Park and Peterson, 2006). The short version VIA-Youth (VIA Youth-96), used in this study, was developed based on the original 198-item VIA-Youth by selecting four items per scale with the highest corrected item-total correlations. The internal consistency of the English version VIA Youth-96 was 0.69–0.93 across scales. The VIA Institute also provides a Chinese version VIA Youth-96, but it is still considered “in development” as the reliability and validity of this translation were unclear.

The original article of Park and Peterson (2006) revealed a four-factor model of the VIA-Youth. Subsequent studies mostly revealed similar, but not identical, solutions to the original article, generating either a four (McGrath and Walker, 2016) or five-factor (Gillham et al., 2011; Toner et al., 2012; Ruch et al., 2014) solution [see Wagner and Ruch (2022) for a review]. Recently, Jabbari et al. (2021) replicated the original four-factor model using the Farsi version VIA-Youth. Yet, Van Eeden et al. (2008) found that the South African version VIA-Youth is more unidimensional than multi-dimensional. The structure of the Chinese version VIA Youth-96 was unknown. One possible reason for the discrepancy in the literature is that the high-order structure of CSs, like other characters, is still evolving from unity to differentiation throughout adolescence and therefore is not stable enough (Shubert et al., 2019). From this perspective, a factor analysis of the Chinese version VIA Youth-96 could shed some light on the possible commonality of CSs during adolescence.

Similar to other personality traits, CSs were proposed to be relatively stable but flexible enough to allow for further development (Peterson and Seligman, 2004). Research on the six-month temporal stability of the VIA-Youth has obtained significant test-retest correlations of around 0.60 (Park and Peterson, 2006). The relational developmental system (RDS) theory posits that development results from interactive, relational processes between individuals and their contexts that unfold over time and individuals (Overton, 2015). Shubert et al. (2019) applied RDS and the orthogenetic principle to character development and found character structure proceeded from being largely diffuse, which was global in late childhood and more differentiated across adolescence. Specifically, in elementary school, children often have overly positive views of their competencies, while when they enter middle school; the youth will explore a multitude of possible selves and characteristics, which could appear to be a dip in self-evaluation (Harter, 2015). From this sense, a better understanding of the lifespan developmental trajectory of CSs could shed some light on the ongoing efforts of promoting positive youth development (Park, 2004).

Generally, CSs were proposed to be slowly increasing by age, but with a dip during adolescence. A recent meta-analysis on cross-sectional studies revealed a significant age difference in 23 of the 24 CSs across the lifespan (from 10 to 65+ years), with 91% of the effects indicating higher levels of the CSs with age, but two CSs (creativity and zest) showed a dip from young (10–12 years) to middle (13–15 years) adolescence (Heintz and Ruch, 2021). During adulthood, several cross-sectional studies showed that the trajectory of CSs was generally slowly increasing by age, especially after the age of 18 (Linley et al., 2007; Baumann et al., 2020). One longitudinal study investigated changes in CSs of adulthood (mostly middle-aged participants, 27–57 years old) in two samples from German-speaking countries across 3.5 years and found that CSs remained stable during the follow-up period (Gander et al., 2020). During adolescence, two studies noted a dip during late childhood and adolescence (age 10–17), where CSs were negatively associated with age (Ruch et al., 2014; Brown et al., 2020). Studies looking at a narrower age range (e.g., age 12–14), however, revealed mixed results (Park and Peterson, 2003, 2006; Ferragut et al., 2014; Kabakci et al., 2019). Only one longitudinal study, as we know, examined the development of CSs across 3 years during adolescence (from age 12 to 14), and found that CSs remained stable during that period (Ferragut et al., 2014).

Most previous studies of CSs were carried out with samples from Western societies. Although the CSs were proposed to be universal among cultures (Peterson and Seligman, 2004), it is unclear if there are cultural differences development trajectory of CSs. Given that CSs can be influenced by contextual factors like culture and parenting (Park and Peterson, 2003; Peterson and Seligman, 2004), it is interesting to ask if the same pattern would be evident in Eastern countries like China. Another issue is that it is unclear if the age-CSs relationship during adolescence is linear or non-linear. Especially, if the “dip” during adolescence end before 17 years old, a u-shape curve (i.e., a quadratic function) would fit the data better than a straight line.

Parenting plays a crucial role in fostering good character in youths (Park, 2004). Among factors related to parenting, parental autonomy support and psychological control have been documented as two of the most important factors that influence youths' development and functioning (Deci and Ryan, 2000). According to the self-determination theory, autonomy is one of the three basic psychological needs (the other two being competence and relatedness) that is essential and universal nutrient for psychological growth and adjustment (Deci and Ryan, 2000). Parental autonomy support refers to parenting behavior of respect and satisfaction for children's need for autonomy, e.g., parents' empathy and respect for children's ideas and feelings, and to supporting the children's independent expression and decision (Grolnick et al., 1997). Parental psychological control is essentially an autonomy-thwarting parenting dimension, referred to as parents' regulation of children's feelings and thoughts (Barber et al., 2005). Parental autonomy support was related to many psychological and educational benefits such as better emotional wellbeing, lower depression, and fewer problem behaviors (e.g., depression, anxiety, delinquent, and aggressive behavior) (Grolnick et al., 1991; Pomerantz and Wang, 2009; Griffith and Grolnick, 2014; Vrolijk et al., 2020). Moreover, perceived parental psychological control was related to maladjustment and even psychopathology in adolescents, including depressive and anxiety symptoms, anxiety, and low self-esteem (Barber, 1996; Pettit et al., 2001; Soenens et al., 2005). One recent study found that parental autonomy support was associated with greater grit (a personality trait involving perseverance and passion for long-term goals in the face of adversity) in emerging adults (Lan et al., 2019). However, it is unclear if and how parental autonomy support and psychological control would affect the changes of CSs in adolescents.

This study aimed to preliminary examine the psychometric properties of the Chinese version VIA Youth-96, and examine the age-related changes in CSs among adolescents. We also assessed the impact of parental autonomy support and psychological control on age-related changes. Based on previous studies, we propose the following hypotheses: (1) all CSs would associate with higher life satisfaction and self-efficacy. (2) CSs would generally decrease during adolescence, the age-CSs association could either be linear or non-linear. (3) Parental autonomy support would slow down the slope of decreasing, while parental psychological control may accelerate it. These hypotheses were not preregistered.

## Method

### Participants

The sample consisted of 959 adolescents between the ages of 10 and 17 (mean age = 12.41 ± 2.08, 49.5% boys) recruited from primary and middle schools in Luzhou and Chengdu city of China from December 2018 to December 2019. The number of respondents in each age group was: 189, 192, 210, 131, 75, 37, 58, and 67 for 10–17 years, respectively. All participants were Han Chinese. As the sample size was not determined a priori, to determine the minimal detectable effect size of our sample, a sensitivity analysis was conducted using G^*^Power 3.1 (Lakens, 2022). The results indicated that with α = 0.05 set as a significance threshold, our final sample size (*N* = 959) was sufficient to detect the effects of *r* = 0.09 for correlations with a statistical power of 0.8.

### Measures

VIA Inventory for Youth (VIA-Youth). VIA-Youth is a self-report measure of CSs for youth ages 10–17 (Park and Peterson, 2006). The Chinese version VIA Youth-96 was used in this study (https://www.viacharacter.org/researchers/assessments/via-youth-96). The scale consists of 96 items, each rated on a Likert scale of five points (from 1 = not like me at all to 5 = very much like me). These items questionnaire assesses 24 CSs among youth. The internal consistency of the Chinese VIA Youth-96 subscales was 0.54–0.86 in this study (Table 1).

**Table 1:** The internal consistency and factor loadings of the Chinese version VIA Youth-96.

	**Cronbach's α**	**McDonald's omega**	**General factor**	**Temperance**	**Intellectual**	**Theological**	**Other-Directed**
ABE	0.78	0.79	0.70		0.33		
Bravery	0.78	0.79	0.82				0.31
Creativity	0.81	0.82	0.62		0.65		
Curiosity	0.76	0.76	0.68		0.54		
Fairness	0.76	0.76	0.85		0.10		
Forgiveness	0.80	0.80	0.80			0.04	
Gratitude	0.66	0.70	0.77			0.13	
Honesty	0.71	0.74	0.76	0.05			
Hope	0.75	0.75	0.74			0.37	
Humility	0.54	0.54	0.59				0.15
Humor	0.86	0.86	0.54			0.54	
Judgment	0.82	0.82	0.80		0.28		
Kindness	0.65	0.67	0.77				0.21
Leadership	0.82	0.82	0.58			0.48	
Love	0.74	0.74	0.74			0.30	
LOL	0.86	0.86	0.72		0.47		
Perseverance	0.80	0.80	0.81	0.27			
Perspective	0.79	0.80	0.74			0.43	
Prudence	0.77	0.77	0.75	0.26			
SR	0.70	0.70	0.67	0.22			
SI	0.71	0.71	0.75			0.37	
Spirituality	0.71	0.72	0.66			0.38	
Teamwork	0.81	0.81	0.89				0.11
Zest	0.78	0.78	0.68			0.50	
Correlations*							
Temperance					0.87	0.86	0.83
Intellectual						0.91	0.85
Theological							0.86

*ABE*, Appreciation of beauty and excellence; *LOL*, love of learning; *SR*, self-regulation; *SI*, social intelligence; *Pearson's correlations calculated based on the total score of each factor.

Satisfaction with Life Scale (SWLS). SWLS is a five-item scale where people judge whether their life is satisfying (Diener et al., 1985). It uses a seven-point scale (from 1 = strongly disagree to 7 = strongly agree). The internal consistency of the scale was 0.81 for this research.

General Self-Efficacy Scale (GSE). GSE consists of 10 items using a four-point Likert-style format (from 1 = strongly disagree to 4 = strongly agree) (Schwarzer and Jerusalem, 1995). The internal consistency of GSE in this study was 0.88.

Perceived Parental Autonomy Support Scale (*P*-PASS). *P*-PASS is a 24-item self-report scale that assesses precepted parental autonomy support and psychological control (Mageau et al., 2015). Twelve items measure the perceptions of autonomy-supportive behaviors. The remaining 12 items measure the perception of psychological control behaviors. Participants rated items in terms of how applicable each statement was to their relationship with their parents (mother and father were rated separately) on a Likert-type scale from 1 (do not agree at all) to 7 (very strongly agree). In this study, the internal consistency for parental autonomy support and psychological control was both 0.95.

### Procedure

The questionnaire was introduced and distributed by teachers in the class. The teacher in school helped in introducing and asking adolescents and their guardians if they were interested in participating. Youths were instructed to fill in the Chinese version VIA Youth-96, along with *P*-PASS, SWLS, and GSE at school, with their parents filling in demographic information at home. All responses were made through an online survey platform (wjx.cn, https://www.wjx.cn/). Participants were not paid for their participation but received individual feedback on their strengths profile, which was sent through E-mail. Informed consent was collected from all participants through the first item after a brief introduction about the survey, where participants were instructed to select one of the two options if they want to continue the survey or opt out. This study was carried out by the Declaration of Helsinki and was approved by the Institutional Review Board of Southwest Medical University.

### Data analysis

#### Structural validity and reliability analyses

The data were analyzed using SPSS 22.0 (SPSS Inc., Chicago, IL, USA) and R version 3.5.3 (R Core Team, 2015). For item analysis, we calculated corrected item-total correlations of each item with its corresponding subscales. Then, to examine the internal consistency of the Chinese version VIA Youth-96, we calculated Cronbach's α and McDonald's omega for each subscale (McDonald, 1999). Following previous literature on CFA (e.g., Little et al., 2002), the unidimensionality of each subscale (based on the items) was examined, using the lavaan package (Rosseel, 2012), before aggregating the items in terms of CS scores.

To assess if the original four-factor model (Park and Peterson, 2006) applies to the Chinese VIA Youth-96, confirmative factor analysis (CFA) was carried out using the lavaan package (Rosseel, 2012). A bifactor model, with four specific factors according to the four-factor model (i.e., temperance strengths, intellectual strengths, theological strengths, and other-directed strengths) and a general factor that was hypothesized to account for the commonality of items, was specified and tested. To justify the inclusion of a general factor, we also specified another four-factor model without the general factor and compared these two models. We considered acceptable model fit as the Tucker–Lewis index (TLI) and the comparative fit index (CFI) > 0.90 (Bentler, 1990), and root mean square error of approximation (RMSEA) < 0.10 (MacCallum et al., 1996). The Akaike information criterion (AIC) and the Bayesian information criterion (BIC) were considered to compare the models, and the lowest value was considered the most appropriate. In the bifactor model, internal consistency is affected by both specific and general variance (Rodriguez et al., 2016); thus, to aid the interpretation of the total and subscale scores, we calculated omega hierarchical (ω_*h*_) and hierarchical subscale (ω_*hs*_). Coefficient ω_*h*_ indicates the variance attributed to the single general factor, while ω_*hs*_ reflects the reliability of a subscale score after controlling for the variance due to the general factor (Reise et al., 2013).

Moreover, to examine the criterion validity of the Chinese VIA Youth-96, correlations between CSs and life satisfaction and general self-efficacy were calculated using Pearson's correlation. The statistical threshold was set at *p* < 0.002 (0.05/24 Bonferroni corrected).

#### Age-related changes in CSs

To determine which one of the linear and non-linear curves could better fit the CSs-age relationship, curve estimations were performed using the fitting linear models function in *R*. For each of the 24 CSs subscale scores, a linear and quadratic model was specified with age as the independent variable, respectively. An extra sum-of-squares *F*-test (Motulsky and Christopoulos, 2004) was performed to compare fit indices between linear and non-linear models.

The correlations between CSs and age were also evaluated with the Spearman correlation, as age was discrete data. Correlations between CSs and parental autonomy support and psychological control were evaluated using Pearson's correlation. Statistical threshold was set at *p* < 0.002 (0.05/24 Bonferroni corrected).

#### Moderation analyses

To test if the age-related changes in CSs would be moderated by parental autonomy support and psychological control, moderation analyses were performed using Model 1 of PROCESS (Hayes, 2013). In total, 48 (24 CSs as the dependent variables; two moderators, i.e., parental autonomy support and psychological control) moderation models were specified. For each model, the score of each CS was entered as the dependent variable, with age as the predictor, parental autonomy support, and psychological control were entered as the moderator. All variables used to create the interaction had centered prior to the analysis in PROCESS. Statistical inference of the moderation effects was carried out using bootstrap estimation, 95% confidence intervals for the age-moderator interaction effect that do not include zero indicate a significant moderation effect at *p* < 0.05. A statistical threshold of 0.002 (0.05/24 Bonferroni corrected) was also adopted for the moderation analysis, given that multiple comparisons were performed.

## Results

### Structural validity and reliability of the Chinese version VIA Youth-96

Four items showed corrected item-total correlations below 0.2, (i.e., VIA-7, 0.17; VIA-30, 0.06; VIA-42, −0.19, VIA-44, 0.16), indicating poor item internal consistency (Streiner and Norman, 2008). These four items were deleted from the scale. The Chinese version VIA Youth-96 hence has 92 valid items, which were used in further analyses. For all subscales, Cronbach's α and McDonald's omega both ranged from 0.54 to 0.86 (Table 1). All CSs showed an acceptable fit to the unidimensionality model (Table S1).

**Figure 1:**
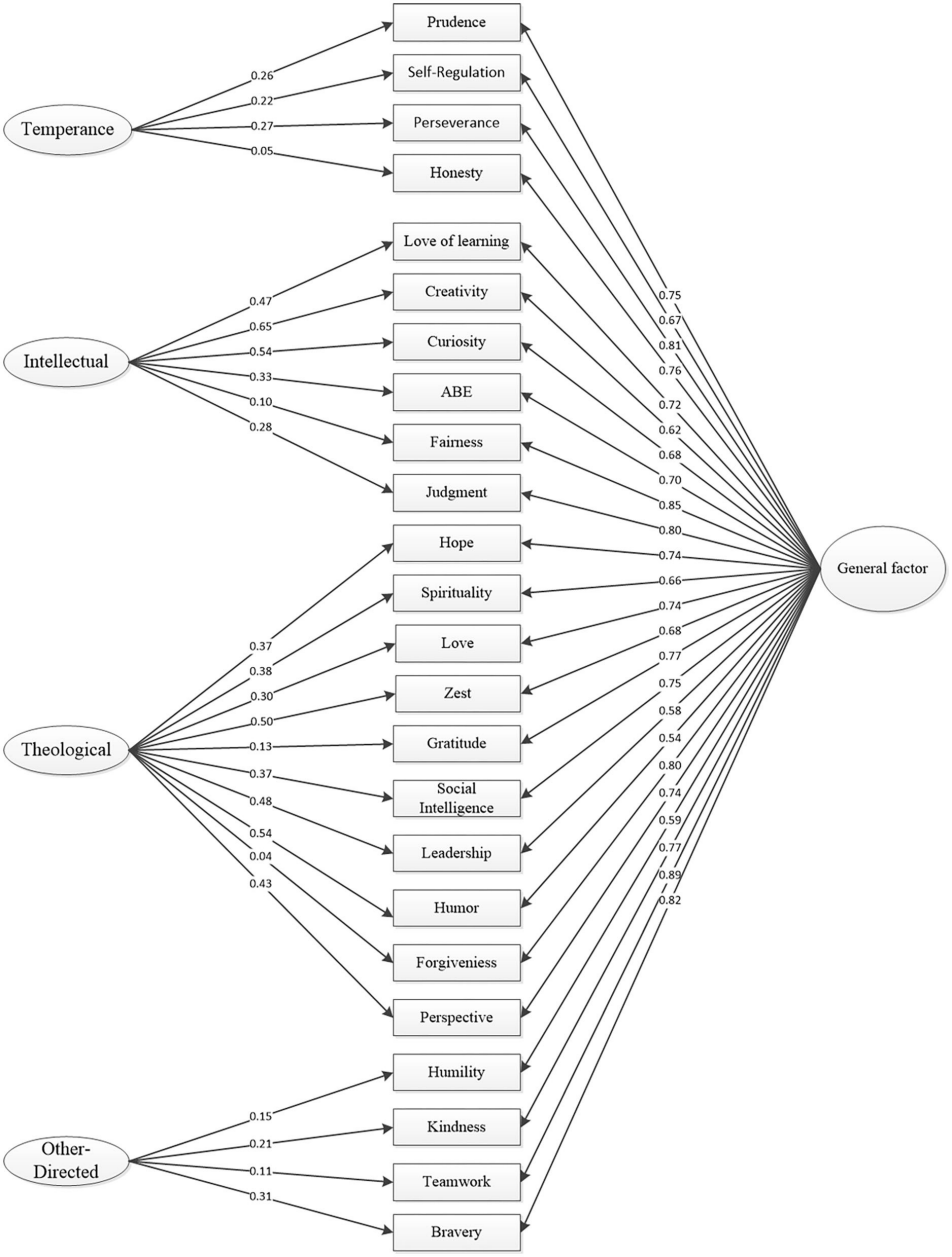
Bifactor model of the Chinese version VIA Youth-96.

The bifactor model with four specific factors and one general factor structure showed adequately fit (χ2 = 1,330.70, df = 222, RMSEA = 0.072, 95%CI [0.068, 0.076], TLI = 0.934, and CFI = 0.947) (Figure 1). Moreover, the bifactor model with a general factor fit the data better than the one without the general factor (AIC:105,689.67 vs. 106,464.42, BIC: 106,069.21 vs. 106,727.18). Further reliability analysis revealed an ω_*h*_ = 0.83 for the general factor. The ω_*hs*_ estimates corresponding to the four specific factors were 0.05, 0.22, 0.21, and 0.05, respectively.

We interpreted the magnitude of the effect sizes based on the guidelines by Funder and Ozer (2019), with *r* = 0.05, 0.10, 0.20, 0.30, and 0.40 corresponding to very small, small, medium, large, and very large effects, respectively. Table 2 shows that there were significant positive correlations between all the CSs and life satisfaction with small to large effect (median *r* = 0.28, range = [0.15, 0.38], *p* < 0.001) and general self-efficacy with large to very large effect (median *r* = 0.48, range = [0.36, 0.58], *p* < 0.001). In sum, the Chinese version VIA Youth-96 showed good criterion validity.

**Table 2:** Correlation results.

	**Age** ^**a**^		**Life satisfaction** ^**b**^		**General self–efficacy** ^**b**^		**Autonomy support** ^**b**^		**Psychological control** ^**b**^	
	***r***	**95%CI**	***r***	**95%CI**	***r***	**95%CI**	***r***	**95%CI**	***r***	**95%CI**
ABE	−0.11*	[−0.174, −0.053]	0.26*	[0.199, 0.337]	0.45*	[0.387, 0.501]	0.36*	[0.295, 0.421]	−0.08	[−0.144, −0.007]
Bravery	−0.12*	[−0.187, −0.063]	0.27*	[0.202, 0.335]	0.48*	[0.425, 0.531]	0.36*	[0.296, 0.413]	−0.06	[−0.136, 0.002]
Creativity	−0.10	[−0.163, −0.028]	0.30*	[0.232, 0.357]	0.54*	[0.487, 0.585]	0.31*	[0.246, 0.367]	0.02	[−0.057, 0.086]
Curiosity	−0.14*	[−0.199, −0.072]	0.29*	[0.224, 0.355]	0.55*	[0.494, 0.590]	0.35*	[0.288, 0.410]	−0.01	[−0.076, 0.060]
Fairness	−0.13*	[−0.184, −0.059]	0.27*	[0.205, 0.345]	0.48*	[0.425, 0.530]	0.39*	[0.334, 0.451]	−0.07	[−0.138, 0.001]
Forgiveness	−0.10	[−0.159, −0.032]	0.24*	[0.171, 0.304]	0.39*	[0.329, 0.444]	0.33*	[0.268, 0.389]	−0.07	[−0.145, −0.009]
Gratitude	−0.07	[−0.031, −0.010]	0.28*	[0.214, 0.352]	0.42*	[0.358, 0.473]	0.39*	[0.321, 0.450]	−0.19*	[−0.257, −0.123]
Honesty	−0.14*	[−0.197, −0.082]	0.29*	[0.226, 0.359]	0.44*	[0.385, 0.496]	0.37*	[0.310, 0.426]	−0.12*	[−0.186, −0.055]
Hope	−0.07	[−0.132, −0.003]	0.29*	[0.222, 0.359]	0.51*	[0.451, 0.557]	0.35*	[0.289, 0.410]	−0.09	[−0.167, −0.019]
Humility	0.02	[−0.040, 0.082]	0.15*	[0.085, 0.226]	0.36*	[0.291, 0.420]	0.22*	[0.157, 0.284]	−0.02	[−0.101, 0.050]
Humor	−0.02	[−0.078, 0.049]	0.20*	[0.136, 0.267]	0.44*	[0.381, 0.488]	0.27*	[0.207, 0.336]	0.00	[−0.066, 0.068]
Judgment	−0.06	[−0.118, 0.008]	0.26*	[0.193, 0.329]	0.51*	[0.464, 0.564]	0.37*	[0.308, 0.425]	−0.06	[−0.124, 0.006]
Kindness	−0.08	[−0.136, −0.011]	0.23*	[0.161, 0.296]	0.44*	[0.376, 0.489]	0.31*	[0.250, 0.376]	−0.04	[−0.115, 0.027]
Leadership	−0.06	[−0.123, 0.006]	0.28*	[0.219, 0.341]	0.51*	[0.455, 0.566]	0.33*	[0.275, 0.390]	0.02	[−0.060, 0.091]
Love	−0.14*	[−0.202, −0.076]	0.38*	[0.314, 0.439]	0.49*	[0.429, 0.535]	0.49*	[0.429, 0.542]	−0.14*	[−0.210, −0.068]
LOL	−0.11*	[−0.170, −0.049]	0.32*	[0.259, 0.370]	0.49*	[0.440, 0.544]	0.38*	[0.315, 0.432]	−0.06	[−0.128, 0.011]
Perseverance	−0.12*	[−0.175, −0.055]	0.32*	[0.253, 0.385]	0.51*	[0.456, 0.558]	0.40*	[0.347, 0.458]	−0.09	[−0.160, −0.021]
Perspective	−0.06	[−0.126, 0.000]	0.31*	[0.245, 0.370]	0.58*	[0.532, 0.632]	0.40*	[0.349, 0.458]	−0.04	[−0.107, 0.038]
Prudence	−0.02	[−0.078, 0.048]	0.23*	[0.162, 0.303]	0.50*	[0.443, 0.551]	0.36*	[0.307, 0.419]	−0.06	[−0.125, 0.008]
SR	−0.04	[−0.109, 0.020]	0.28*	[0.208, 0.341]	0.43*	[0.378, 0.487]	0.32*	[0.266, 0.377]	−0.04	[−0.102, 0.024]
SI	0.00	[−0.062, 0.065]	0.30*	[0.230, 0.365]	0.50*	[0.444, 0.555]	0.37*	[0.310, 0.424]	−0.05	[−0.119, 0.015]
Spirituality	−0.08	[−0.147, −0.017]	0.30*	[0.236, 0.365]	0.48*	[0.424, 0.532]	0.36*	[0.302, 0.417]	0.03	[−0.047, 0.096]
Teamwork	−0.08	[−0.139, −0.010]	0.26*	[0.190, 0.326]	0.46*	[0.403, 0.514]	0.36*	[0.365, 0.474]	−0.12*	[−0.187, −0.047]
Zest	−0.12*	[−0.176, −0.053]	0.35*	[0.291, 0.418]	0.50*	[0.445, 0.547]	0.42*	[0.294, 0.414]	−0.06	[−0.131, 0.007]

*p < 0.002 (0.05/24, Bonferroni corrected). *ABE*, Appreciation of beauty and excellence; *LOL*, love of learning; *SR*, self-regulation; *SI*, social intelligence. ^a^calculated using Spearman's correlation, ^*b*^calculated using Pearson's correlation.

### The age-related changes in CSs among adolescents

**Figure 2:**
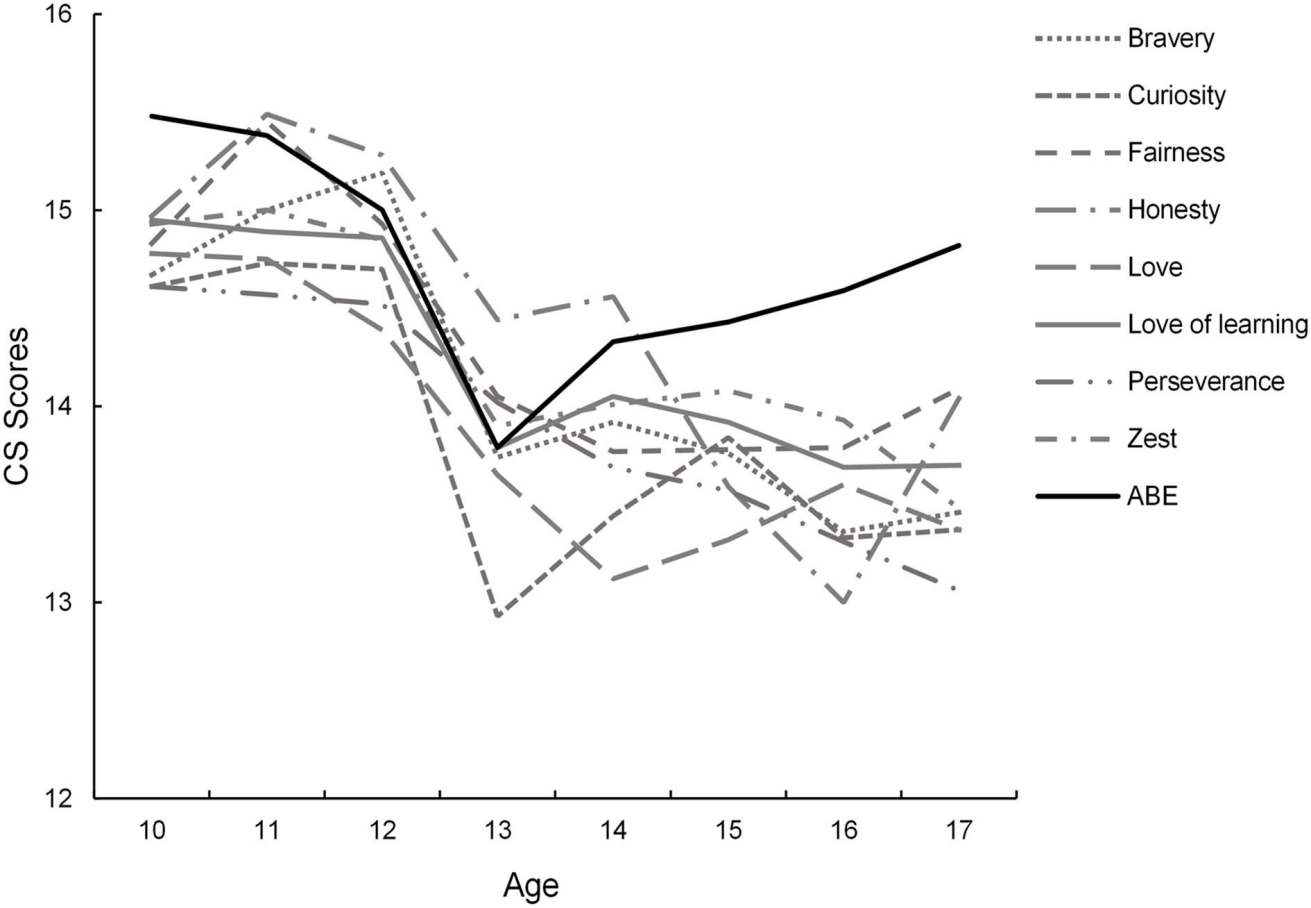
The age-related changes in CSs. Only CSs showed significant age-related changes were presented, including appreciation of beauty and excellence (ABE), bravery, curiosity, fairness, honesty, love, love of learning, perseverance, and zest.

According to the curve estimation analyses, linear and non-linear models showed a similar fit for 23/24 of the CSs (all *p* > 0.18). The only exception was the “appreciation of beauty and excellence,” which was better described by a quadratic curve (AIC: 5360.37 vs. 5355.92, *F* = 6.45, *p* = 0.011). For the 23 CSs, linear regressions were performed with scores of each CS as the dependent variable and age as the independent variable, separately. The results showed that eight of the 23 CSs were significantly negatively predicted by age, namely bravery (β = −0.13, *p* < 0.001), curiosity (β = −0.13, *p* < 0.001), fairness (β = −0.12, *p* < 0.001), honesty (β = −0.15, *p* < 0.001), love (β = −0.14, *p* < 0.001), love of learning (β = −0.11, *p* = 0.001), perseverance (β = −0.13, *p* < 0.001), and zest (β = −0.12, *p* < 0.001) (Figure 2). For the appreciation of beauty and excellence, a quadratic regression was performed. The quadratic regression revealed a significant effect of age on the appreciation of beauty and excellence (*F*_(2, 956)_ = 6.93, *p* = 0.001, β_1_ = −2.09, β_2_ = 0.07), indicating that the score of this CS would first go down and rebound when past the bottom point (Figure 2). Similarly, Spearman correlations analyses revealed negative correlations between most CSs and age with very small effect (median r = −0.08, range = [−0.14, 0.02]) (Table 2). Nine out of the 24 CSs (the same CSs that showed significant changes in linear/non-linear regressions, r = −0.14 to −0.11, *p* < 0.002) reached a significant level after Bonferroni correction (0.05/24), with small effect size.

### Parental autonomy support and psychological control influencing of CSs

In line with our hypothesis, there were significant positive correlations between all CSs and parental autonomy support with medium to very large effect (median *r* = 0.36, range = [0.22, 0.49], *p* < 0.001). On the contrary, psychological control was with very small to small negatively correlations with CSs (median *r* = −0.06, range = [−0.19, 0.03]), four correlations reached a significant level with small effect after Bonferroni correction (*r* = −0.19 to −0.12, *p* < 0.002) (Table 2).

### Moderation effects of parental autonomy support and psychological control on age-CS associations

**Figure 3:**
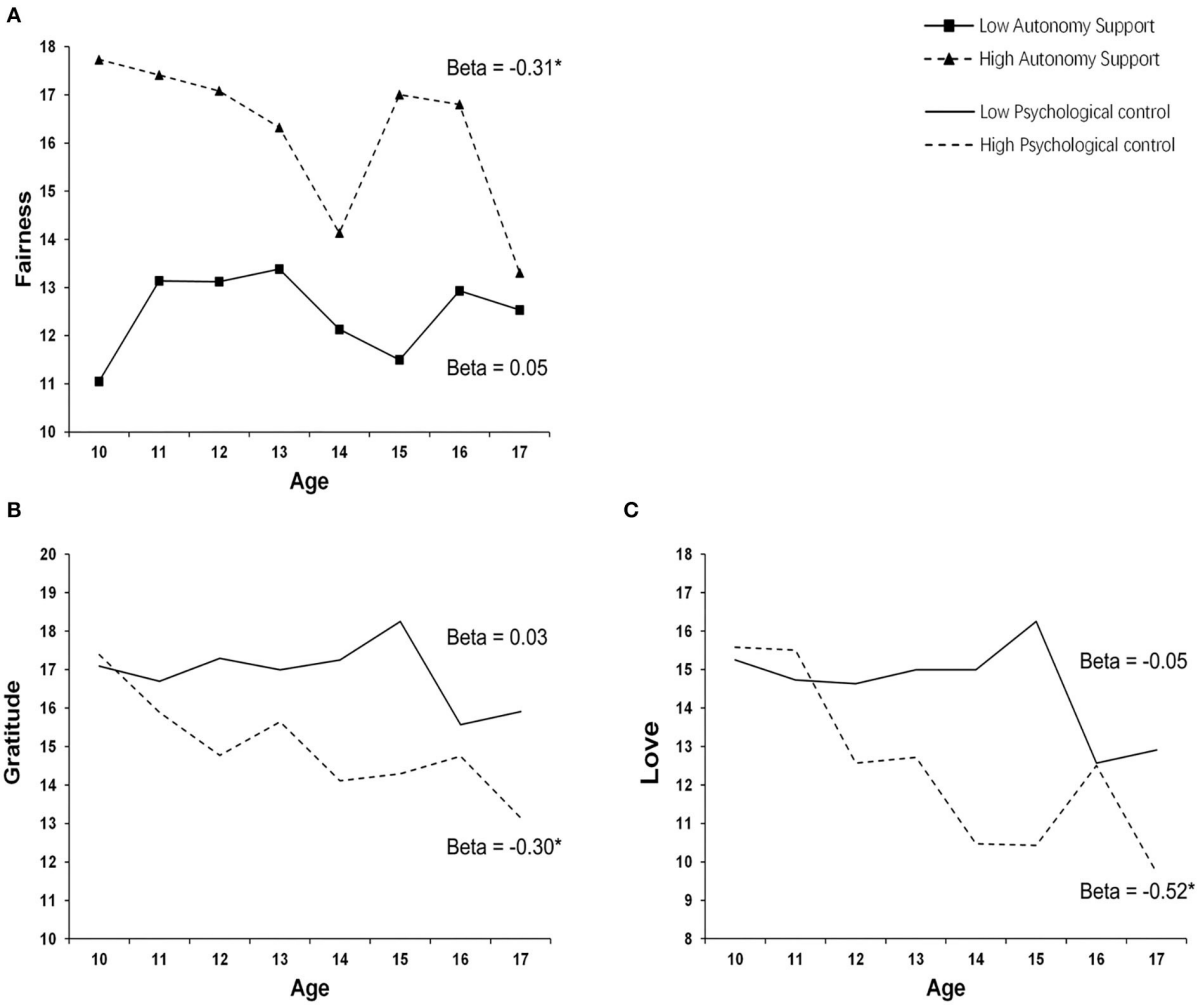
Results of moderation analyses. Parental autonomy support has a significant effect on age-CSs associations in fairness **(A)**. Psychological control has a significant effect on age-CSs associations in gratitude **(B)** and love **(C)**.

Significant interactions were identified: parental autonomy support moderating the effect of age on fairness (*F* = 10.53, *b* = −0.0120, *p* = 0.0012), psychological control moderating the effect of age on gratitude (*F* = 10.16, *b* = −0.0099, *p* = 0.0015), and love (*F* =13.82, *b* = −0.0140, *p* = 0.0002). Simple slope analyses indicated scores of fairness significantly decreased by age in participants with higher parental autonomy support (β = −0.31, 95%CI = [−0.47, −0.15], *p* < 0.001), but not in those with lower parental autonomy support. Similarly, scores of gratitude (β = −0.30, 95%CI = [−0.45, −0.16], *p* < 0.001) and love (β = −0.52, 95%CI = [−0.69, −0.34], *p* < 0.001) significantly decreased by age in participants with higher parental psychological control, but not in those with lower parental psychological control (Figure 3).

## Discussion

This study provided reliability and validity of the Chinese version VIA Youth-96 and tested age-related changes in CSs. The Chinese version VIA Youth-96 showed a good fit for the original four-factor structure, and CS scores were significantly correlated with life satisfaction and self-efficacy, indicating a good structural and criterion validity of the scale. The internal consistency was 0.54–0.86 for subscales. Moreover, this study revealed significant age-related changes in CSs among adolescents, eight CSs significantly linearly declined by age.

Our CFA confirmed a good fit of the original four-factor model for the Chinese version VIA Youth-96. This result was in line with the study of English (Park and Peterson, 2006) and Farsi version VIA Youth-96 (Jabbari et al., 2021), suggesting that the four-factor model may be applicable in multiple cultures, including the Chinese context. Further reliability analysis, however, revealed high ω_*h*_ (0.83) but low ω_*hs*_ (0.05–0.22) of the four-factor structure, indicating that the vast majority of reliable variance is attributable to a single common source, rather than the specific factors. The existence of a general factor highlights the commonality of items and indicates that there could be a common basis for all CSs and the four specific factors are not reliable enough. This explanation also reflects the unity-to-differentiation pattern of character development across adolescence (Shubert et al., 2019). In short, given the low reliability of specific factors, applying the four-factor model to this scale requires extra caution.

In the second part of this study, we examined the relationship between age and CSs in the present sample (covering an age range from 10 to 17 years) and found that eight of the CSs were linearly decreased across age. Our curve estimation showed that almost all (23/24) CSs were decreasing linearly by age till 17 years old. This result was in line with the findings of Ruch et al. (2014) and Brown et al. (2020), where CSs scores were linearly decreased with age during adolescence. Additionally, the sample of these two studies was composed of adolescents from many cultures [125 countries for Brown et al. (2020) and two countries for Ruch et al. (2014)]; our results together with these findings suggest that the linear decline of CSs during adolescents could be identical cross-cultures (Ruch et al., 2014). Importantly, the specific CSs (i.e., bravery, curiosity, fairness, honesty, love, love of learning, perseverance, and zest) showing a linear decrease in our results were identical to the findings of Brown et al. (2020). Just as RDS theory suggests that development is the result of an interactive relational process that unfolds over time and personally between an individual and their environment, character constructs proceed from a global state to become increasingly differentiated with age (Werner, 1957; Overton, 2015). For adolescents, their surroundings become more complex with age, and adolescents may feel uncertain about their ability to navigate mature social environments and therefore perceive themselves as incapable of demonstrating these strengths. In addition, the declining pattern of CSs in the current results also agreed with the disruption hypothesis of personality development which proposes that the biological, social, and psychological transitions during adolescence were accompanied by a temporary decline in some aspects of personality maturity (Soto and Tackett, 2015). Our findings may shed some light on the development of interventions to enhance CSs in adolescents.

This study revealed a close association between CSs and parental autonomy support and psychological control. Particularly, autonomy support was positively correlated with CSs, while psychological control showed the opposite effect. These results in the present study largely correspond with previous findings that autonomy support is related to the superior psychological development of youth, while psychological control does the opposite (Grolnick et al., 1991; Barber, 1996; Pettit et al., 2001; Soenens et al., 2005; Pomerantz and Wang, 2009; Griffith and Grolnick, 2014; Vrolijk et al., 2020), particularly the findings of autonomy support associated with a greater level of character among late adolescents and early adults (Lan et al., 2019). Note that, the correlation between autonomy support and CSs (median *r* = 0.36, range = [0.22, 0.49]) was higher than between CSs and age (median *r* = −0.08, range = [−0.14, 0.02]), suggesting that perceived parental autonomy support has the potential to alter the effect of age on CSs. According to the self-determination theory, the need for autonomy must be satisfied for individuals to experience healthy growth and development, and parents are the main socializing agents in youth life (Lekes et al., 2010). Parental autonomy support is, therefore, an important factor in the development of children and adolescents. Our results hence highlight that parental autonomy support (and maybe lower psychological control) could provide a basis for encouraging personal CSs to grow (Lavy, 2019).

Our moderation analyses revealed a significant impact of parental autonomy support and psychological control on the association between age and CSs. The moderation effects indicated that particular CSs (namely fairness, gratitude, and love) were decreased by age only in the case of high autonomy support or high psychological control; otherwise, they would remain relatively stable across adolescence. High autonomy support and high psychological control hence seem necessary to maintain the “normal” (declining) trajectory of changes in CSs. The result that high parental psychological control seems necessary for youth to exhibit a “normal” developmental trajectory of CSs may seem counterintuitive, as parental psychological control was usually related to “bad” psychological consequences (Barber, 1996; Pettit et al., 2001; Soenens et al., 2005). These results may be accounted for by the East–West culture differences in the attitude toward parental control. There was evidence that a certain degree of parental control could be tolerable and beneficial for Chinese youth, but not for Western youth (Grusec et al., 1997). Note that, although the moderator effects were significant after correction, our samples were relatively small for an analysis considering statistical interactions (Gelman et al., 2020). Therefore, these effects need to be interpreted with caution, and replication of this finding is necessary.

The research has some limitations. First, this study was a cross-sectional study, a longitudinal design is needed to examine the stability of CSs in future studies. Second, the sample size of this study is relatively small, which could limit the generalizability of our conclusions. Third, the test–retest reliability of the Chinese version VIA Youth-96 was not assessed; further studies on the temporal reliability of the scale are warranted. Finally, only the VIA Youth-96 was tested in this study, our results thus should not be applied to the full version (the 198-item version) VIA-Youth.

## Conclusion

The current study confirmed a four-factor structure, good criterion validity, and largely acceptable internal consistency of the Chinese version VIA Youth-96. The finding supports the use of the Chinese version VIA Youth-96 for the assessment of CSs among Chinese youth. This study also provided clear evidence for a pattern of declining CSs by age from 10 to 17, and that parental autonomy support and psychological control significantly moderated the association between age and CSs. These findings should contribute meaningfully to further research and provide critical information for parents and those who are interested in the intervention of adolescents' CS.

## Data availability statement

The original contributions presented in the study are publicly available. This data can be found here: https://osf.io/mp5rw/.

## Ethics statement

The studies involving human participants were reviewed and approved by Institutional Review Board of Southwest Medical University. Written informed consent to participate in this study was provided by the participants' legal guardian/next of kin.

## Author contributions

XC and SX: Formal analysis and writing the original draft. WL: Conceptualization, methodology, writing, reviewing, and editing. WL and YH: Funding acquisition. JC: Writing, reviewing, editing, investigation, resources, and project administration. CQ, YH, MT, XL, and XZ: Investigation. KL and BX: Writing, reviewing, and editing. All authors contributed to the article and approved the submitted version.

## Funding

This work was supported in part by the National Undergraduate Training Program for Innovation and Entrepreneurship (Grant No. 201816032170); the Sichuan Education Department Research Project (Grant No. 18ZB0634); and the University Mental Health Education Training Base program of the Sichuan Education Department (Grant No. 2020SXJP016).

## Conflict of interest

The authors declare that the research was conducted in the absence of any commercial or financial relationships that could be construed as a potential conflict of interest.

## Publisher's note

All claims expressed in this article are solely those of the authors and do not necessarily represent those of their affiliated organizations, or those of the publisher, the editors and the reviewers. Any product that may be evaluated in this article, or claim that may be made by its manufacturer, is not guaranteed or endorsed by the publisher.
