# Supplementary material for: Initial validation of the Chinese version VIA Youth-96 and age-related changes in character strengths among adolescents
Source: Front Psychol. 2022 Oct 11;13:906171. doi: 10.3389/fpsyg.2022.906171 (PMC9595728; doi:10.3389/fpsyg.2022.906171)
Supplement: Supplementary file 1 [file Table_1.DOCX]

|  | χ2 | df | GFI | CFI | NFI | IFI | RMSEA |
| --- | --- | --- | --- | --- | --- | --- | --- |
| ABE | 10.777 | 2 | 0.999 | 0.992 | 0.990 | 0.992 | 0.068 |
| Bravery | 0.589 | 2 | 1.000 | 1.000 | 0.999 | 1.001 | 0.000 |
| Creativity | 4.393 | 2 | 1.000 | 0.998 | 0.997 | 0.998 | 0.035 |
| Curiosity | 4.516 | 2 | 1.000 | 0.997 | 0.995 | 0.997 | 0.036 |
| Fairness | 0.014 | 2 | 1.000 | 1.000 | 1.000 | 1.002 | 0.000 |
| Forgiveness | 0.000 | 0 | 1.000 | 1.000 | 1.000 | 1.000 | 0.000 |
| Gratitude | 4.489 | 2 | 1.000 | 0.996 | 0.994 | 0.996 | 0.036 |
| Honesty | 18.147 | 2 | 0.999 | 0.983 | 0.981 | 0.983 | 0.092 |
| Hope | 7.345 | 2 | 1.000 | 0.994 | 0.992 | 0.992 | 0.053 |
| Humility | 25.531 | 2 | 0.998 | 0.917 | 0.911 | 0.918 | 0.111 |
| Humor | 7.590 | 2 | 0.999 | 0.997 | 0.996 | 0.997 | 0.054 |
| Judgment | 9.741 | 2 | 0.999 | 0.994 | 0.993 | 0.994 | 0.064 |
| Kindness | 1.340 | 2 | 1.000 | 1.000 | 0.998 | 1.001 | 0.000 |
| Leadership | 4.383 | 2 | 1.000 | 0.998 | 0.997 | 0.998 | 0.035 |
| Love | 24.641 | 2 | 0.998 | 0.972 | 0.970 | 0.972 | 0.109 |
| LOL | 41.199 | 2 | 0.997 | 0.979 | 0.978 | 0.979 | 0.143 |
| Perseverance | 15.946 | 2 | 0.999 | 0.988 | 0.986 | 0.988 | 0.085 |
| Perspective | 8.877 | 2 | 0.999 | 0.994 | 0.992 | 0.994 | 0.060 |
| Prudence | 0.000 | 0 | 1.000 | 1.000 | 1.000 | 1.000 | 0.000 |
| SR | 0.000 | 0 | 1.000 | 1.000 | 1.000 | 1.000 | 0.000 |
| SI | 8.363 | 2 | 0.999 | 0.991 | 0.988 | 0.991 | 0.058 |
| Spirituality | 5.336 | 2 | 1.000 | 0.996 | 0.993 | 0.996 | 0.042 |
| Teamwork | 9.388 | 2 | 1.000 | 0.994 | 0.992 | 0.994 | 0.062 |
| Zest | 8.012 | 2 | 0.999 | 0.994 | 0.992 | 0.994 | 0.056 |

**Table S1.** Model fit statistics for the unidimensional model of the 24 CSs

*CSs*, Character strengths, *ABE*, Appreciation of Beauty and Excellence, *LOL*, Love of learning, *SR*, Self-Regulation, *SI*, Social Intelligence.
